# Case report: Improving quality of life through hyaluronic acid-based fillers after orbital cancer treatment

**DOI:** 10.3389/fonc.2024.1501556

**Published:** 2024-12-18

**Authors:** Sara Egidi, Valentino Valentini, Amalia Schiavetti

**Affiliations:** ^1^ Private Practice, Rome, Italy; ^2^ Maxillofacial Surgery Department, Sapienza University, Rome, Italy; ^3^ Department of Pediatrics, Sapienza University, Rome, Italy

**Keywords:** dermal filler, hyaluronic acid, aesthetic medicine, post-surgical complication, myoepithelial carcinoma

## Abstract

**Background:**

Myoepithelial carcinoma is a very rare yet aggressive tumor in children. Surgical intervention and local radiotherapy often lead to post-therapy complications, affecting both the aesthetic and functional quality of life in survivors. Hyaluronic acid (HA) dermal fillers offer a minimally invasive option to improve the appearance and quality of life for these patients once they are declared tumor-free.

**Case presentation:**

We present the case of an 18-year-old girl with a history of myoepithelial carcinoma in the right upper orbit, diagnosed at the age of 8. The patient underwent surgery to remove the tumor and lacrimal gland, followed by chemotherapy and radiotherapy. A complete response to treatment was achieved, and the patient was monitored with regular clinical and radiological exams for 5 years, after which she was declared tumor-free and followed for late effects of therapy. Post-surgical radiotherapy resulted in atrophy of the upper orbital frame and functional complications. The patient exhibited upper eyelid retraction, ptosis, continuous lacrimation, and conjunctival redness. Ten years after treatment, the patient underwent dermal filler injections using Aliaxin^®^ Essential Volume (A_EV_) and Aliaxin^®^ Superior Volume (A_SV_) to address the aesthetic impairment of the upper right orbit. A_SV_ was administered using a 22G x 50mm cannula on the periosteum of the superior orbital frame, entering from the outer canthus. A_EV_ was injected with a cannula into the muscle, also entering from the outer canthus. Before treatment, the patient exhibited upper eyelid retraction, ptosis, continuous lacrimation, and conjunctival redness. Following the injections, improvements were observed in all pre-treatment symptoms. The closing ability of the upper eyelid was restored, along with superior orbital volume and symmetry. Enhanced eyelid function improved eye hydration, reduced redness in the conjunctiva, and led to better vision and overall quality of life.

**Conclusion:**

To our knowledge, this is the first reported case of using dermal fillers to treat ocular changes resulting from cancer treatment. Injections of A_EV_ and A_SV_ provided both aesthetic and functional improvements.

## Introduction

Dermal fillers, particularly hyaluronic acid (HA)-based formulations, are widely recognized for their effectiveness in aesthetic medicine. However, their application in post-oncological settings remains underexplored. Traditional reconstructive approaches after oncological surgery often involve invasive techniques, yet there is a growing interest in minimally invasive procedures that restore both aesthetics and function. Recent advancements in cross-linked HA fillers, such as BDDE-cross-linked gels, offer significant soft tissue augmentation with minimal complications (1). Despite these advances, the use of such fillers to correct deformities following tumor treatments, especially in pediatric and young adult cancer survivors, is under-documented.

Improvements in early cancer detection and treatment have significantly increased survival rates, with 5-year survival rates for children aged 0-14 now exceeding 80% (1–3). As survival rates rise, maintaining quality of life post-treatment becomes paramount, as many patients live with long-term effects from the disease and its treatment (2). Surgery, chemotherapy, and radiotherapy can cause chronic conditions, including scarring, skin changes, and damage to mucosal barriers, often resulting in altered physical appearance (2, 4–8). These complications can be particularly severe for childhood cancer survivors, where functional and cosmetic issues involving muscle and soft tissues are common. Ocular and visual complications, for example, often arise from surgery and radiotherapy. Ocular malignancies pose unique challenges due to long-term aesthetic and functional changes in structures like the eyelid and lacrimal gland (5). Myoepithelial carcinoma is a very rare but aggressive tumor that can occur in the orbital region, although this is extremely rare (9–11). The multimodal treatment approach, including surgery, chemotherapy, and radiotherapy, demands expertise from a multidisciplinary team to balance optimal results with minimizing side effects (11, 12). Surgery often leads to noticeable changes in periocular tissues, and radiation in the head and neck may further exacerbate complications such as dry eye syndrome and corneal damage (12).

Aesthetic treatments, including dermal fillers, botulinum neurotoxins (BoNTA), thread lifts and laser therapies, are increasingly being used to improve the quality of life in cancer patients experiencing physical changes (1, 13). However, it is crucial to ensure these treatments do not interfere with ongoing cancer therapies or exacerbate immunosuppression. Although highly effective, surgical operations or the use of thread lifts are not the first treatment choice for all the patients, as they require anesthetic procedures and can be considered as invasive approaches. On the other hand, the use of BoNTA can represent an effective and non-invasive approach (14). However, the duration is relatively short requiring applications every 3-5 months and the injection requires a high knowledge of facial anatomy to avoid any risk of potential complications (14). For patients requesting non-invasive procedures, dermal fillers, such as HA, methylcellulose, and calcium hydroxyapatite are the most widely used and guarantee long-lasting results (12-18 months after treatment) (15). However, semi-permanent fillers (e.g. calcium hydroxyapatite) could display a higher rate of inflammatory reactions, such as nodules or granulomas due to their biostimulatory mechanisms, which involves an immune-mediated response (16). Although they are considered as temporary products, HA-based fillers are effective and safe products due to their biocompatibility and potentially low immunogenic properties. HA, a naturally occurring linear polysaccharide, plays a role in various biological processes depending on its molecular size, including anti-inflammatory actions and regulation of the extracellular dermal matrix (15, 17). The biophysical and rheological properties of HA fillers, such as concentration, molecular weight, G prime (G’), and Tan delta, can be customized for different clinical applications (18–20). The Aliaxin^®^ line of fillers, consisting of BDDE-cross-linked HA hydrogels, is commercialized by IBSA Farmaceutici Italia Srl (17). Each formulation has distinct properties due to its unique composition of molecular weights and degree of cross-linking (18–23). For example, Aliaxin^®^ Essential Volume (A_EV_) and Aliaxin^®^ Superior Volume (A_SV_) are characterized by the highest degree of cross-linking, making them ideal for creating volume and lifting effects (24, 25). In this report, we demonstrate the successful use of BDDE-cross-linked HA gels for correcting post-oncological orbital defects. This innovative approach not only restores aesthetic outcomes but also addresses functional impairments, such as eyelid retraction and dryness. To our knowledge, this is the first documented case of HA gel use for correcting orbital deformities following lacrimal gland tumor resection in a cancer survivor, highlighting the potential role of dermal fillers in oncological rehabilitation.

## Case report

**Figure 1:**
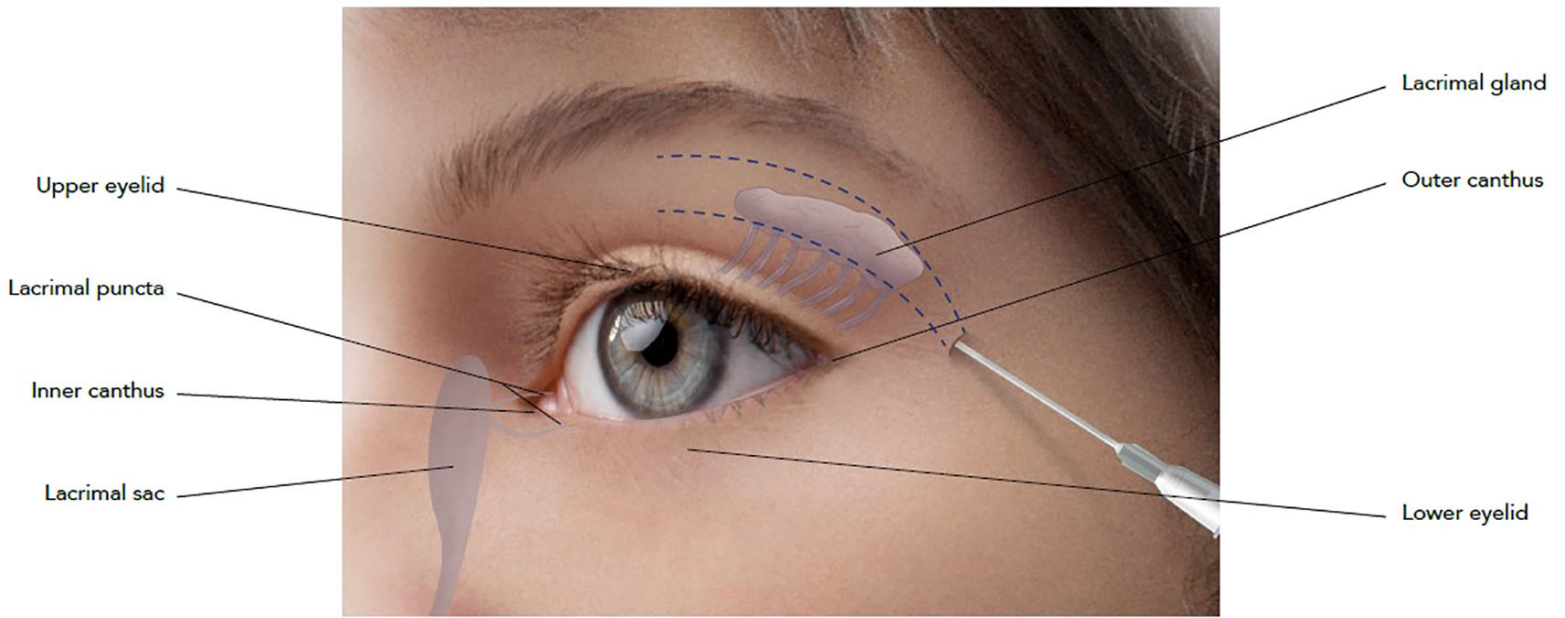
Schematic representation of injection technique. Cannula entry-point has been identified at the outer canthus of the eye and injection has been performed using a 22G x 50 mm cannula. A_SV_ has been applied on the periosteum of the superior orbital frame, while A_EV_ has been injected on the muscle layer.

An 8-year-old female with no significant medical history presented with a 1 cm mass in the superior region of the right orbit, exhibiting no other symptoms. She was seen at the Pediatric Oncology Department of Sapienza University of Rome. A complete excision with clear margins was performed at the Maxillofacial Surgery Department, Sapienza University (Rome, Italy), followed by histological analysis. The diagnosis was lymphoepithelioma-like carcinoma, and no additional treatment was given. Five months later, the patient experienced a local recurrence. Radiological assessments, including ultrasound, Magnetic Resonance Imaging (MRI), and 18F-fluorodeoxyglucose-positron emission tomography (FDG-PET), revealed a 5 cm mass in the superior right orbit with muscle invasion. To reduce tumor size and enable a more conservative surgical approach, the patient was treated with the ICE chemotherapy regimen: ifosfamide (900 mg/m², days 1-5), cisplatin (20 mg/m², days 1-5), and etoposide (60 mg/m², days 1-5). After two courses of chemotherapy, MRI confirmed partial tumor response. A complete eye-sparing surgical excision of the orbital mass was then performed. Histological revision and molecular genetic testing identified the tumor as myoepithelial carcinoma with an EWSR1 rearrangement. Patient underwent radiotherapy to the right orbit (41 Gy), followed by four courses of IVE chemotherapy (ifosfamide 3 g/m², days 1-3; vincristine 1.5 mg/m², day 1; etoposide 150 mg/m², days 1-3) and two courses of VAC chemotherapy (cyclophosphamide 1,500 mg/m², vincristine 1.5 mg/m², adriamycin 40 mg/m²). The treatment led to a complete response, and the patient was monitored with clinical and radiological examinations, in particular magnetic resonance imaging (MRI) every six months for five years, after which she was declared tumor-free and followed for late effects of therapy. Post-surgical radiotherapy resulted in atrophy of the upper orbital frame and cicatricial defects. The removal of the lacrimal gland required the patient to use artificial tears for lubrication. At 18 years of age, in December 2018, the patient underwent treatment with Aliaxin^®^ to address the orbital structural changes caused by tumor removal. BDDE-cross-linked hyaluronic acid (HA) gels were chosen due to their volumizing properties and minimal risk of migration or adverse effects. Specifically, Aliaxin^®^ fillers have been selected due to their rheological characteristics, such as the high cohesivity values, long-term duration and good safety profile, which ensures a respectful and natural approach for the patient (23). The injection was performed with a 22G x 50 mm cannula to allow precise placement along the periosteum and muscle layers, minimizing the risk of intravascular injection, which is crucial in the orbital region due to the risk of vascular complications (26). The treatment aimed to improve both the function and appearance of the eye structures following surgery. Aliaxin^®^ Superior Volume (A_SV_) was injected via a 22G x 50 mm cannula along the periosteum of the superior orbital frame, entering from the outer canthus. Aliaxin^®^ Essential Volume (A_EV_) was injected into the muscle layer, also entering from the outer canthus (**Figure 1**). A_SV_ and A_EV_ were selected due to their high molecular weight and cross-linking, which provided the desired volumizing and cohesive properties for soft tissue augmentation. A_SV_ restored the structural framework of the orbit, while A_EV_ improved the mechanical function of the eyelid through soft tissue support. A total of 1 mL of each product was used, and the entire treatment took 15 minutes.

**Figure 2:**
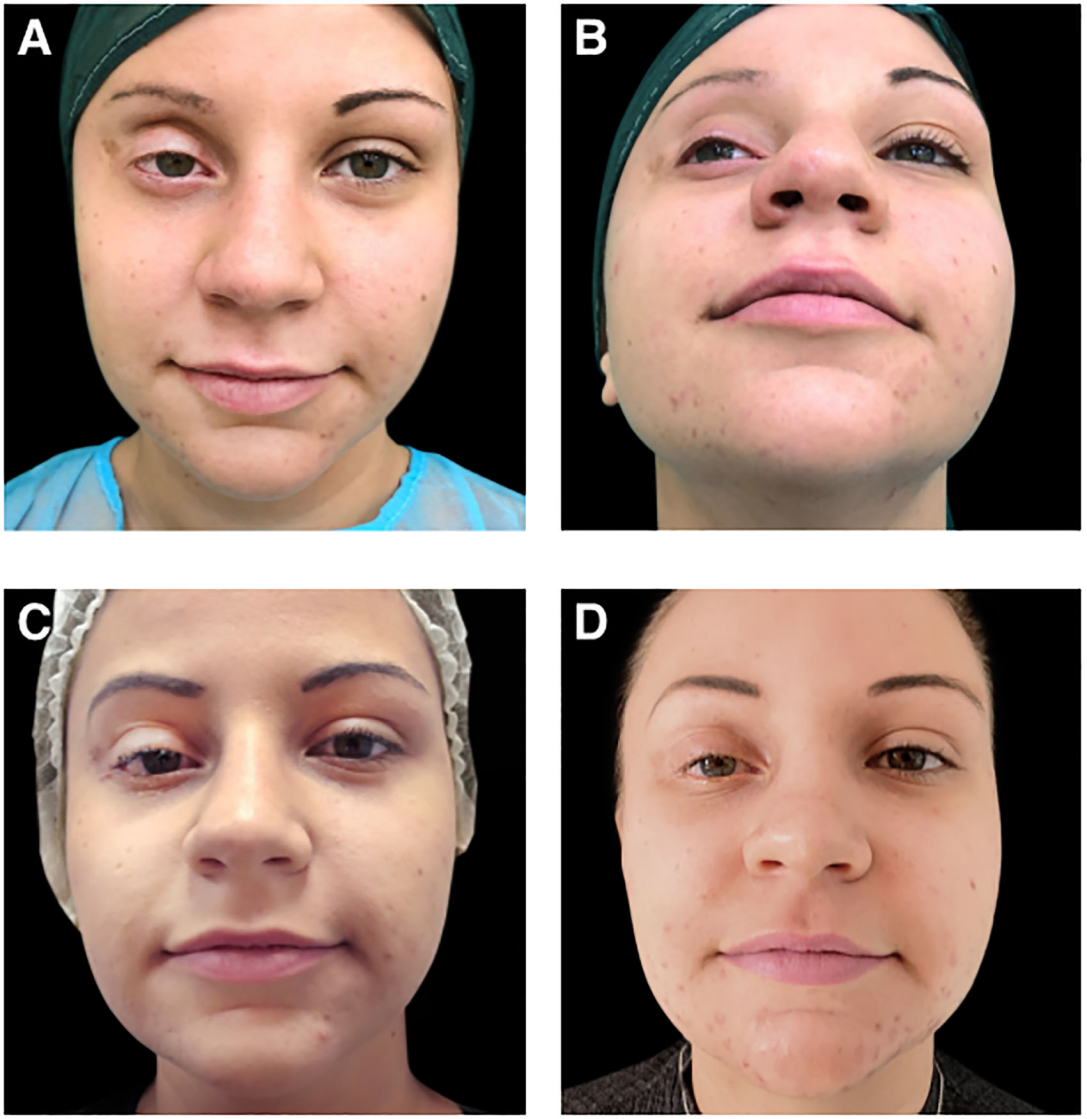
Photographs of the patient: **(A, B)** before aesthetic treatment; **(C)** 1-month after treatment with Aliaxin^®^ Essential Volume and Aliaxin^®^ Superior Volume; **(D)** 14-months after treatment. Patient provided informed consent for the use of these images for scientific research.

Before treatment with Aliaxin^®^, the patient presented with upper eyelid retraction due to a lack of orbital fat tissue, upper eyelid ptosis, continuous lacrimation, and redness of the conjunctiva (**Figures 2A, B**). Clinical improvements were assessed by the investigator post-treatment and during follow-up visits at 1 month and 14 months after injection (**Figures 2C, D**). Additionally, patient quality of life and vision were evaluated using the EYE-Q (Effects of Youngsters’ Eyesight on Quality of Life) questionnaire at baseline (before treatment) and at the follow-up visits. The EYE-Q questionnaire, designed for children aged 8 and older and young adults (up to 18 years), includes 26 items that assess vision functionality using a 5-point scale: 1 (excellent), 2 (good), 3 (fair), 4 (poor), 5 (very poor), and 6 (blind). It also examines symptoms related to common uveitis (eye redness, blurry vision, eye pain, and photosensitivity) and the patient’s feelings about different aspects of daily life (10). Three key scores were calculated: the total vision score (EYE-Q Total), the visual function score (EYE-Q VF), and the vision-related quality of life score (EYE-Q VRQL). At the 1-month follow-up, and more notably at the 14-month follow-up, the patient demonstrated significant improvement in all pre-treatment symptoms. Discomfort was reduced, upper eyelid ptosis improved, and swelling of the lower eyelid decreased (**Figure 2D**). The ability to close the upper eyelid improved, and the volume and symmetry of the superior orbital frame were restored. These changes enhanced eye hydration, leading to a reduction in conjunctival redness. Both the patient and the investigator reported improvements in vision at 1 month and 14 months post-treatment. Furthermore, the patient reported marked improvements in total vision score, visual functionalities, and vision-related quality of life at each follow-up visit, as measured by the EYE-Q questionnaire (**Table 1**). The treatment with Aliaxin^®^ A_EV_ and A_SV_ provided both aesthetic and functional benefits.

**Table 1:** Quality of life and vision functionalities assessed using EYE-Q questionnaire at baseline (before treatment) and 1 and 14 months after treatment.

	Baseline	1 month after treatment	14 months after treatment
EYE-Q Total	3.3	2.1	2.5
EYE-Q VF	3.55	2.75	3.05
EYE-Q VQRL	2.9	2	2.1

EYE-Q, Eyesight on Quality of Life; EYE-Q VF, EYE-Q visual function score; EYE-Q VQRL, EYE-Q vision-related quality of life score.

## Discussion

Dermal fillers provide a non-surgical, minimally invasive option for correcting aesthetic deformities in patients following cancer treatment. A key advantage of HA-based fillers, particularly those cross-linked with BDDE, is their ability to offer long-lasting volumizing effects while maintaining excellent biocompatibility. The elastic properties of BDDE-cross-linked HA gels make them especially suitable for dynamic areas like the eyelids, where both volume and movement are critical for functional outcomes. This case report demonstrates the use of Aliaxin^®^ A_EV_ and A_SV_ dermal fillers to address specific post-surgical sequelae after cancer treatment, improving both the aesthetic and functional aspects of orbital soft tissues and muscles. Notably, there was significant improvement in superior orbital frame volume and restoration of symmetry in the treated eye. This also led to improvements in eyelid ptosis, muscle function, and eye hydration. A_EV_ and A_SV_ fillers are highly elastic, capable of recreating volume in the upper eyelid with a prolonged effect, without the adverse events typically associated with fillers, such as nodule formation (25–27). Limitations of this case report include the relatively short-period follow-up. Moreover, this kind of approach are also to be considered as temporary, and patient could also require other treatments to improve the aesthetic and functional impairment. An extended follow-up period is therefore required to fully evaluate fillers approaches. However, in this case the treatment remained effective for over a year post-injection. The sustained mechanical improvement in the upper eyelid, coupled with enhanced eye hydration, contributed to a decrease in conjunctival redness and an improvement in vision 14 months after treatment. These changes positively impacted the patient’s quality of life.

Although aesthetic treatments can represent an effective and useful approach to ameliorate patients’ quality of life and impairments due to post-surgical sequelae due to cancer treatment, there are crucial medico-legal and ethical evaluations to be considered. Although rare, fillers could lead to potential adverse events and complications, such as oedema, bruising, swelling, lumps or nodules, granulomas and inflammatory nodules (27–29). Another potential severe complication is represented by hypersensitivity reactions to dermal fillers; these can be classified into acute, which occurs within minutes to hours after injection, and delayed, which can take place from 24 hours to months after treatment (30). In particular, several articles recently published have also highlighted the overall increase of hypersensitivity reactions associated with fillers after the COVID-19 pandemic (31, 32). These aspects need to be considered and evaluated by healthcare practitioners, especially for the treatment of fragile patients, such as post-cancer ones. Therefore, it is essential that healthcare practitioners possess a full overview of patients’ anamnesis and history as well as a deep knowledge of anatomy, injection guidelines and associated risks. It is also fundamental for the patient to receive an informed consent form which is not merely need to obtain a signed consent, but it is focused to provide a complete explanation and understanding of the proposed products, treatments and the associated risks (14).

For this case report, it is essential to highlight that this patient had been in remission for 10 years post-oncological treatment and has been declared tumor-free. Moreover, the patient did not report any adverse events and/or hypersensitivity reaction during the 14 months follow-up period. However, patients with active disease, those still undergoing cancer treatment, or those who are psychologically unstable are not recommended candidates and should not be treated with dermal fillers (2, 33). Physicians must assess each patient’s suitability for aesthetic procedures on a case-by-case basis, considering both their physical and emotional well-being in collaboration with a multidisciplinary team (2).

Despite the importance of physical appearance for cancer survivors, there are limited studies investigating the use of dermal fillers in this population (34). One small study explored the use of small gel particle HA with lidocaine dermal filler in combination with abobotulinumtoxinA in post-chemotherapy patients, reporting well-tolerated treatments and significant aesthetic benefits after eight weeks (13). Another study successfully corrected soft tissue loss secondary to facial lymphoma using HA and poly-L-lactic acid (35). While studies in non-cancer patients have consistently reported positive quality of life changes following the use of minimally invasive cosmetic injectables (36, 37), the application of dermal fillers in cancer survivors requires careful consideration.

Though the risk of adverse events such as inflammation or infection is generally low, the long-term safety of dermal fillers in patients with a history of radiation or chemotherapy is not yet fully understood. It is essential to exclude patients with active disease or those undergoing immunosuppressive therapy from treatment, as their ability to heal and manage infections may be compromised (33).

Considering these aspects and the multiple clinical, cultural and ethical challenges of the aesthetic medicine nowadays, it is also crucial to work with a multidisciplinary approach, ensuring the respect of a pertinent ethical conduct for all the patients (38).

A thorough multidisciplinary approach, combined with patient education, ensures that both the physical and emotional needs of cancer survivors are carefully addressed when considering aesthetic interventions.

## Conclusion

To our knowledge, this is the first reported use of dermal fillers to address ocular changes following tumor resection and radiotherapy. Treatment with A_SV_ and A_EV_ provided both aesthetic and functional improvements, enhancing the patient’s vision and overall quality of life. The successful application of BDDE-cross-linked hyaluronic acid fillers in this case introduces a novel approach to post-oncological rehabilitation. This case highlights the potential of dermal fillers to restore both the aesthetic appearance and functional capacity of the upper eyelid after orbital surgery, showcasing their versatility beyond traditional cosmetic use.

With the growing population of cancer survivors facing long-term complications, minimally invasive treatments like dermal fillers present a promising therapeutic option. However, further research is necessary to validate these findings in larger patient cohorts and to evaluate long-term outcomes, particularly in patients with complex oncological histories. By presenting this case, we aim to encourage the integration of aesthetic treatments into oncological aftercare, where quality of life remains a central concern.

All procedures performed in studies involving human participants were in accordance with the ethical standards of the institutional and/or national research committee and with the 1964 Helsinki declaration and its later amendments or comparable ethical standards.

## Data Availability

The data presented in this case report are available on request from the corresponding author. The data are not publicly available due to privacy and ethical reasons.

## References

[1] Proietti I Skroza N Mambrin A Marraffa F Tolino E Bernardini N. Aesthetic treatments in cancer patients. Clin Cosmet Investig Dermatol. (2021) 14:1831–7. doi: 10.2147/CCID.S342734 PMC8654687 34898993

[2] American Cancer Society. Cancer Treatment & Survivorship Facts & Figures 2022-2024. Atlanta: American Cancer Society (2022).

[3] Board PPTE. Late effects of treatment for childhood cancer (PDQ^®^). In: PDQ Cancer Information Summaries. National Cancer Institute (US (2004).

[4] Asencio-Durán M Fernández-Gutiérrez E Larrañaga-Cores M Klein-Burgos C Dabad-Moreno JV Capote-Díez M. Ocular side effects of oncological therapies: Review. Arch Soc Esp Oftalmol (Engl Ed). (2023) 99(3):109–32. doi: 10.1016/j.oftale.2023.11.003 37949110

[5] Balasubramanian A Kannan NS. Eyelid Malignancies- always quite challenging. J Clin Diagn Res. (2017) 11:Xr01–xr4. doi: 10.7860/JCDR/2017/23695.9582 PMC5427420 28511494

[6] Bronner AK Hood AF. Cutaneous complications of chemotherapeutic agents. J Am Acad Dermatol. (1983) 9:645–63. doi: 10.1016/S0190-9622(83)70177-5 6643764

[7] Hu SC Hou MF Luo KH Chuang HY Wei SY Chen GS. Changes in biophysical properties of the skin following radiotherapy for breast cancer. J Dermatol. (2014) 41:1087–94. doi: 10.1111/jde.2014.41.issue-12 25354814

[8] Kang D Kim IR Im YH Park YH Ahn JS Lee JE. Quantitative changes in skin composition parameters due to chemotherapy in breast cancer patients: a cohort study. Breast Cancer Res Treat. (2015) 152:675–82. doi: 10.1007/s10549-015-3502-4 26198993

[9] Hornick JL Fletcher CD. Myoepithelial tumors of soft tissue: a clinicopathologic and immunohistochemical study of 101 cases with evaluation of prognostic parameters. Am J Surg pathology. (2003) 27:1183–96. doi: 10.1097/00000478-200309000-00001 12960802

[10] Gleason BC Fletcher CD. Myoepithelial carcinoma of soft tissue in children: an aggressive neoplasm analyzed in a series of 29 cases. Am J Surg Pathol. (2007) 31:1813–24. doi: 10.1097/PAS.0b013e31805f6775 18043035

[11] Bisogno G Tagarelli A Schiavetti A Scarzello G Ferrari A Cecchetto G. Myoepithelial carcinoma treatment in children: a report from the TREP project. Pediatr Blood Cancer. (2014) 61:643–6. doi: 10.1002/pbc.24818 24136896

[12] Nuzzi R Trossarello M Bartoncini S Marolo P Franco P Mantovani C. Ocular complications after radiation therapy: an observational study. Clin Ophthalmol. (2020) 14:3153–66. doi: 10.2147/OPTH.S263291 PMC7555281 33116366

[13] Shamban A. Safety and efficacy of facial rejuvenation with small gel particle hyaluronic acid with lidocaine and abobotulinumtoxinA in post-chemotherapy patients: A phase IV investigator-initiated study. J Clin Aesthet Dermatol. (2014) 7:31–6. PMC3930538 24563694

[14] Nittari G Savva D Gibelli F Vulcanescu D Leo D Ricci G. Anatomy, etiology, management, and medico-legal implications of botulinum-induced blepharoptosis. Curr Rev Clin Exp Pharmacol. (2024) 20:32–7. doi: 10.2174/0127724328310459240809073519 PMC11826903 39882702

[15] Fallacara A Baldini E Manfredini S Vertuani S. Hyaluronic acid in the third millennium. Polymers (Basel). (2018) 10(7):701. doi: 10.3390/polym10070701 30960626 PMC6403654

[16] Nowag B Schäfer D Hengl T Corduff N Goldie K. Biostimulating fillers and induction of inflammatory pathways: A preclinical investigation of macrophage response to calcium hydroxylapatite and poly-L lactic acid. J Cosmet Dermatol. (2024) 23:99–106. doi: 10.1111/jocd.15928 37593832

[17] La Gatta A De Rosa M Frezza MA Catalano C Meloni M Schiraldi C. Biophysical and biological characterization of a new line of hyaluronan-based dermal fillers: A scientific rationale to specific clinical indications. Mater Sci Eng C Mater Biol Appl. (2016) 68:565–72. doi: 10.1016/j.msec.2016.06.008 27524055

[18] La Gatta A Papa A Schiraldi C De Rosa M. Hyaluronan dermal fillers via crosslinking with 1,4-butandiol diglycidyl ether: Exploitation of heterogeneous reaction conditions. J Biomed Materials Res Part B: Appl Biomaterials. (2016) 104:9–18. doi: 10.1002/jbm.b.v104.1 25611588

[19] La Gatta A Salzillo R Catalano C D’Agostino A Pirozzi AVA De Rosa M. Hyaluronan-based hydrogels as dermal fillers: The biophysical properties that translate into a “volumetric” effect. PloS One. (2019) 14:e0218287. doi: 10.1371/journal.pone.0218287 31185059 PMC6559669

[20] La Gatta A Schiraldi C Zaccaria G Cassuto D. Hyaluronan dermal fillers: efforts towards a wider biophysical characterization and the correlation of the biophysical parameters to the clinical outcome. Clin Cosmet Investig Dermatol. (2020) 13:87–97. doi: 10.2147/CCID.S220227 PMC6995295 32095081

[21] Aliaxin^®^ EV Essential Volume [package insert]. Switzerland: Rose Pharma S.A., Lugano (2019).

[22] Aliaxin^®^ SV Superior Volume [package insert]. Switzerland: Rose Pharma S.A., Lugano (2019).

[23] Molina B Romano D Zazzaron M Kramer E Cigni C Grimolizzi F. Full face tailored treatments using hyaluronan dermal fillers: biophysical characterization for safe and effective approaches. Cosmetics. (2024) 11:144. doi: 10.3390/cosmetics11040144

[24] Sparavigna A La Gatta A Bellia G La Penna L Giori AM Vecchi G. Evaluation of the volumizing performance of a new volumizer filler in volunteers with age-related midfacial volume defects. Clin Cosmet Investig Dermatol. (2020) 13:683–90. doi: 10.2147/CCID.S262839 PMC7502383 32982362

[25] La Gatta A Bedini E Aschettino M Finamore R Schiraldi C. Hyaluronan hydrogels: rheology and stability in relation to the type/level of biopolymer chemical modification. Polymers (Basel). (2022) 14(12):2402. doi: 10.3390/polym14122402 35745978 PMC9228881

[26] Funt D Pavicic T. Dermal fillers in aesthetics: an overview of adverse events and treatment approaches. Clin Cosmet Investig Dermatol. (2013) 6:295–316. doi: 10.2147/ccid.s50546 PMC3865975 24363560

[27] King M Bassett S Davies E King S. Management of delayed onset nodules. J Clin Aesthet Dermatol. (2016) 9:E1–e5. PMC5300719 28210391

[28] Ghareeb FM Hassan MSA El Nahas MA Salem MSES. Complicated facial fillers: management algorithm. Plast Reconstr Surg Glob Open. (2022) 10:e4445. doi: 10.1097/GOX.0000000000004445 35923984 PMC9307301

[29] Cohen JL Patel M Hicks J. Ten-year global postmarket safety surveillance of delayed complications with a flexible cheek filler. Dermatol Surg. (2022) 48:1126–7. doi: 10.1097/DSS.0000000000003587 PMC9521579 36129206

[30] Chung KL Convery C Ejikeme I Ghanem AM. A systematic review of the literature of delayed inflammatory reactions after hyaluronic acid filler injection to estimate the incidence of delayed type hypersensitivity reaction. Aesthet Surg J. (2020) 40:NP286–300. doi: 10.1093/asj/sjz222 31410442

[31] Rowland-Warmann MJ. Hypersensitivity reaction to Hyaluronic Acid Dermal filler following novel Coronavirus infection - a case report. J Cosmet Dermatol. (2021) 20:1557–62. doi: 10.1111/jocd.14074 PMC8251125 33735503

[32] Savva D Battineni G Amenta F Nittari G. Hypersensitivity reaction to hyaluronic acid dermal filler after the Pfizer vaccination against SARS-CoV-2. Int J Infect Dis. (2021) 113:233–5. doi: 10.1016/j.ijid.2021.09.066 PMC8479353 34597761

[33] Rossi AM Hibler BP Navarrete-Dechent C Lacouture ME. Restorative oncodermatology: Diagnosis and management of dermatologic sequelae from cancer therapies. J Am Acad Dermatol. (2021) 85:693–707. doi: 10.1016/j.jaad.2020.08.005 32781177 PMC7868476

[34] Carver CS Smith RG Petronis VM Antoni MH. Quality of life among long-term survivors of breast cancer: Different types of antecedents predict different classes of outcomes. Psychooncology. (2006) 15:749–58. doi: 10.1002/pon.v15:9 16304622

[35] Yan BY Hibler BP Day RA Myskowski PL Rossi AM. Treatment of facial lipoatrophy secondary to subcutaneous panniculitis-like T-cell lymphoma. Dermatol Surg. (2020) 46:702–4. doi: 10.1097/DSS.0000000000001862 30829756

[36] Scharschmidt D Mirastschijski U Preiss S Brähler E Fischer T Borkenhagen A. Body image, personality traits, and quality of life in botulinum toxin A and dermal filler patients. Aesthetic Plast Surg. (2018) 42:1119–25. doi: 10.1007/s00266-018-1165-3 29948095

[37] Sobanko JF Dai J Gelfand JM Sarwer DB Percec I. Prospective cohort study investigating changes in body image, quality of life, and self-esteem following minimally invasive cosmetic procedures. Dermatol Surg. (2018) 44:1121–8. doi: 10.1097/DSS.0000000000001523 29659404

[38] da Prato EB Cartier H Margara A Molina B Tateo A Grimolizzi F. The ethical foundations of patient-centered care in aesthetic medicine. Philos Ethics Humanit Med. (2024) 19:1. doi: 10.1186/s13010-024-00151-1 38317236 PMC10845625

